# Neutralizing antibodies against the SARS-CoV-2 Delta and Omicron variants following heterologous CoronaVac plus BNT162b2 booster vaccination

**DOI:** 10.1038/s41591-022-01705-6

**Published:** 2022-01-20

**Authors:** Eddy Pérez-Then, Carolina Lucas, Valter Silva Monteiro, Marija Miric, Vivian Brache, Leila Cochon, Chantal B. F. Vogels, Amyn A. Malik, Elena De la Cruz, Aidelis Jorge, Margarita De los Santos, Patricia Leon, Mallery I. Breban, Kendall Billig, Inci Yildirim, Claire Pearson, Randy Downing, Emily Gagnon, Anthony Muyombwe, Jafar Razeq, Melissa Campbell, Albert I. Ko, Saad B. Omer, Nathan D. Grubaugh, Sten H. Vermund, Akiko Iwasaki

**Affiliations:** 1 Ministry of Health, Santo Domingo, Dominican Republic; 2 Department of Immunobiology, Yale University School of Medicine, New Haven, CT USA; 3 Two Oceans in Health, Santo Domingo, Dominican Republic; 4 Biomedical Research Department, Profamilia, Santo Domingo, Dominican Republic; 5 Department of Epidemiology of Microbial Diseases, Yale School of Public Health, New Haven, CT USA; 6 Yale Institute for Global Health, Yale University, New Haven, CT USA; 7 Section of Infectious Diseases, Department of Medicine, Yale University School of Medicine, New Haven, CT USA; 8 Laboratorio de Referencia, Santo Domingo, Dominican Republic; 9 Section of Infectious Diseases and Global Health, Department of Pediatrics, Yale University School of Medicine, New Haven, CT USA; 10 Connecticut State Department of Public Health, Rocky Hill, CT USA; 11 Instituto Gonçalo Moniz, Fundação Oswaldo Cruz, Salvador, Brazil; 12 Department of Ecology and Evolutionary Biology, Yale University, New Haven, CT USA; 13 Yale School of Public Health, New Haven, CT USA; 14 Howard Hughes Medical Institute, Chevy Chase, MD USA

**Keywords:** Antibodies, Inactivated vaccines

## Abstract

The recent emergence of the SARS-CoV-2 Omicron variant is raising concerns because of its increased transmissibility and its numerous spike mutations, which have the potential to evade neutralizing antibodies elicited by COVID-19 vaccines. Here we evaluated the effects of a heterologous BNT162b2 mRNA vaccine booster on the humoral immunity of participants who had received a two-dose regimen of CoronaVac, an inactivated vaccine used globally. We found that a heterologous CoronaVac prime vaccination of two doses followed by a BNT162b2 booster induces elevated virus-specific antibody levels and potent neutralization activity against the ancestral virus and the Delta variant, resembling the titers obtained after two doses of mRNA vaccines. Although neutralization of Omicron was undetectable in participants who had received a two-dose regimen of CoronaVac, the BNT162b2 booster resulted in a 1.4-fold increase in neutralization activity against Omicron compared with the two-dose mRNA vaccine. Despite this increase, neutralizing antibody titers were reduced by 7.1-fold and 3.6-fold for Omicron compared with the ancestral strain and the Delta variant, respectively. These findings have immediate implications for multiple countries that previously used a CoronaVac regimen and reinforce the idea that the Omicron variant is associated with immune escape from vaccines or infection-induced immunity, highlighting the global need for vaccine boosters to combat the impact of emerging variants.

## Abstract (web summary)

BNT162b2 booster vaccination in individuals who had previously received two doses of CoronaVac elevates neutralizing antibodies against the Omicron variant, but titers remain reduced compared with those against the ancestral SARS-CoV-2 virus and the Delta variant.

## Main

The ongoing evolution of severe acute respiratory syndrome coronavirus 2 (SARS-CoV-2) and the recent emergence of the Omicron variant raise concerns about its increased transmissibility and vaccine effectiveness. CoronaVac is a two-dose β-propiolactone-inactivated, aluminum hydroxide-adjuvanted coronavirus disease 2019 (COVID-19) vaccine. CoronaVac is widely used globally and has been authorized in 48 countries, with 85% and 80% effectiveness against hospital admission and death, respectively^1^. However, with the emergence of new SARS-CoV-2 variants and the waning immunity of vaccines over time, multiple countries have initiated the use of booster doses^2–4^. The Dominican Republic was among the first countries to recommend a third booster dose to address potential waning immunity and reduced effectiveness against variants.

The Omicron variant contains up to 36 mutations in the spike protein^5^, rendering many vaccines less effective^6,7^. The Omicron variant is also highly transmissible, overtaking Delta as the dominant variant in many countries. To assess the potential risk of vaccine immune evasion we assembled a cohort of CoronaVac-vaccinated individuals who had received a heterologous BNT162b2 mRNA vaccine boost. We investigated vaccine-induced neutralizing antibody titers against the Delta (B.1.617.2) and the Omicron (BA.1 sublineage of B.1.1.529) variants and compared them with the neutralization titers produced against the ancestral A lineage, using authentic SARS-CoV-2 isolates.

To characterize SARS-CoV-2-specific adaptive immune responses we analyzed plasma samples from 101 non-hospitalized adult participants who received the BNT162b2 booster dose between 30 July and 27 August 2021, at least 4 weeks after the second dose of CoronaVac. Plasma samples were collected longitudinally at Departamento de Investigaciones Biomedicas, Clinica Evangelina Rodriguez, Profamilia, Santo Domingo, Dominican Republic, at baseline (prior to the booster), 7 and 28 days after the booster (third dose), and were analyzed with ELISA and neutralization assays using authentic virus. Data from a previous cohort, composed of healthcare workers (HCWs) from the Yale-New Haven Hospital who received two doses of an mRNA COVID-19 vaccine (mRNA-1273, Moderna or BNT162b2, Pfizer-BioNTech) were used as a reference^8^. The mean age of the 101 participants from the Dominican Republic (the majority of whom (70%) were female) was 40.4 (s.d. 13.4) years and the body mass index was 27.6 (s.d. 5.5) kg m^−2^. Cohort demographics, vaccination and infection status are summarized in Extended Data Tables 1 and 2.

**Fig. 1:**
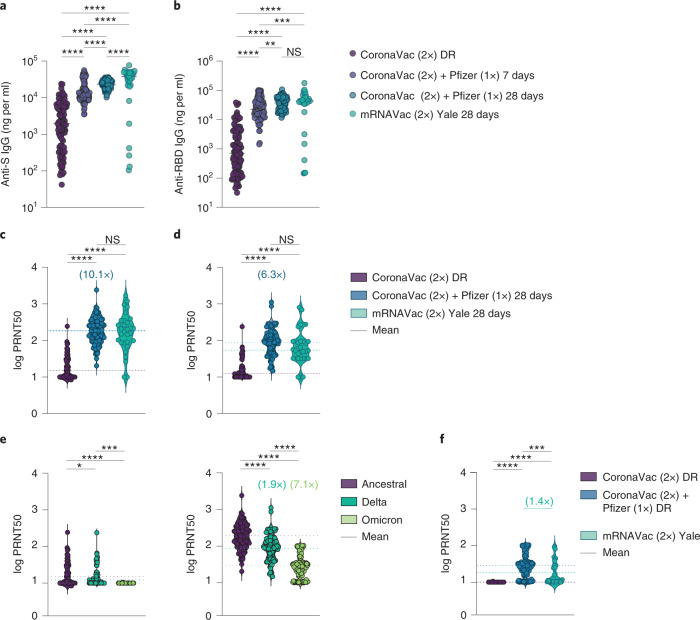
Characterization of vaccine-induced immunity after heterologous CoronaVac–BNT162b2 vaccination. Dominican Republic (DR) participants received two doses of CoronaVac followed by a heterologous booster with BNT162b2 mRNA vaccine. Plasma samples were collected at baseline, before the booster (CoronaVac (2x) DR), and at 7 and 28 days after the booster (CoronaVac (2x) + Pfizer (1x)). The HCW participants from Yale-New Haven Hospital received two doses of the mRNA vaccine and the plasma samples were used for comparison (mRNAVac (2x) Yale). **a**,**b**, Plasma reactivity to the spike (S) protein (**a**) and RBD (**b**) measured with ELISA (DR *n* = 101 and Yale *n* = 32 at each respective time point). Horizontal lines indicate the mean values. **c**–**f**, Neutralization assay using wild-type SARS-CoV-2. **c**,**d**, Plasma neutralization capacity against the ancestral strain (WA1, USA) (**c**) and the Delta variant (**d**) by time (DR, *n* = 101 in each group; Yale, *n* = 32). The numbers in parentheses indicate the median fold change in neutralization resistance for the indicated variants compared with the ancestral strain. **e**, Plasma neutralization capacity against the ancestral strain and the Delta and Omicron variants at baseline (left) and at 28 days after the booster (right) (*n* = 64 for baseline Omicron variant, *n* = 101 in other group). **f**, Plasma neutralization capacity against Omicron by time. CoronaVac (2x) DR, *n* = 64; CoronaVac (2x) + Pfizer (1x) DR, *n* = 101; mRNAVac (2x) Yale, *n* = 32. Significance was assessed with one-way ANOVA corrected for multiple comparisons using Tukey’s method. Violin plots represent the mean ± s.d. Dotted lines indicate the mean and are colored accordingly. Each circle represents a single individual. *P < 0.05; **P < 0.01; ***P < 0.001; ****P < 0.0001; NS, not significant.

Plasma antibody reactivity to the spike protein and receptor binding domain (RBD) of SARS-CoV-2 were measured at baseline and at 7 and 28 days after the BNT162b2 booster. Virus-specific immunoglobulin G (IgG) titers increased at 7 days above baseline, and were further elevated on day 28 after the booster shot (Fig. 1a,b). Individuals who received the CoronaVac prime vaccination of two doses followed by the BNT162b2 booster developed high anti-RBD IgG titers that reached equivalent levels to those of the HCWs who received two doses of mRNA vaccines^8^.

We then measured the ability of serum samples to neutralize SARS-CoV-2, lineage A (ancestral strain, USA-WA1/2020) and lineage B.1.617.2 (Delta variant). Individuals who were fully vaccinated with CoronaVac and who had received the BNT162b2 booster had a 10.1- and 6.3-fold increase in neutralization activity against the ancestral strain and the Delta variant, respectively, 28 days after the booster shot. No statistically significant differences were observed in neutralization titers between individuals who had received the CoronaVac–BNT162b2 regimen and those who had received the two-dose mRNA regimen, 28 days after the last shot (Fig. 1c,d).

**Extended Data Fig. 1:**
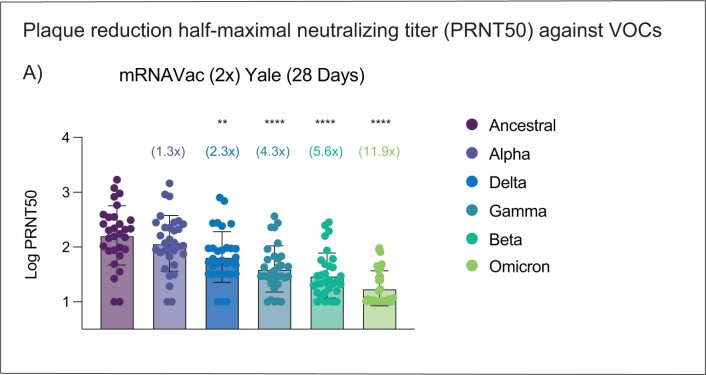
Impact of SARS-CoV-2 Omicron on neutralization capacity of in mRNA-vaccinated participants. Plasma neutralization titers against ancestral lineage A virus, (WA1, USA) and VOCs: Alpha, Beta, Delta and Omicron. SARS-CoV-2 variants were isolated from nasopharyngeal swabs of infected individuals and ancestral (WA1, USA) isolate was obtained from BEI. Neutralization capacity was accessed using plasma samples from HCW participants that received 2 doses of the mRNA vaccine (mRNAVac (2x) Yale), 28 days post second vaccination dose at the experimental sixfold serial dilutions (from 1:3 to 1:2430). Significance was assessed by One-way ANOVA corrected for multiple comparisons using Dunnett’s method. Neutralization capacity to the variants was compared to neutralization capacity against the ancestral strain. Boxes represent mean values ± standard deviations. The numbers in parentheses indicate the median fold change in neutralization resistance for the indicated variants compared to ancestral strain. n = 32/group Each dot represents a single individual. ****p < 0.0001 **p < 0.005.

Next, we extended our analysis to investigate potential neutralizing antibody escape against the Omicron variant after vaccination. In the HCW cohort we observed a 14.5-fold reduction in neutralization titers against the Omicron variant (Extended Data Fig. 1) at 28 days after the second vaccination dose (the peak of neutralization titers^8^), measured by half-maximum plaque reduction neutralizing assays (PRNT50).

Booster doses of homologous mRNA vaccines have been shown to enhance neutralizing antibody response against the Omicron variant^9–14^. To further assess neutralization activity in the heterologous vaccination regimen, we compared neutralization titers against the ancestral virus, the Delta and the Omicron variants before and after the BNT162b2 booster. Omicron was 7.1-fold less sensitive to neutralization than ancestral virus and 3.6-fold less sensitive than the Delta variant when assayed with plasma samples obtained 28 days after the BNT162b2 booster (Fig. 1e). Notably, plasma from those who received two doses of CoronaVac had no neutralizing antibodies to Omicron prior to the BNT162b2 booster (Fig. 1f). After the booster, 80% of CoronaVac–BNT162b2 recipients developed neutralizing antibody titers against the Omicron variant. The BNT162b2 booster resulted in a 1.4-fold increase in neutralization activity against Omicron compared with the two-dose mRNA vaccine, 28 days after the last shot (Fig. 1f). Despite this increase, neutralizing antibody titers were reduced by 7.1-fold and 3.6-fold for Omicron compared with ancestral and Delta SARS-CoV-2, respectively.

**Fig. 2:**
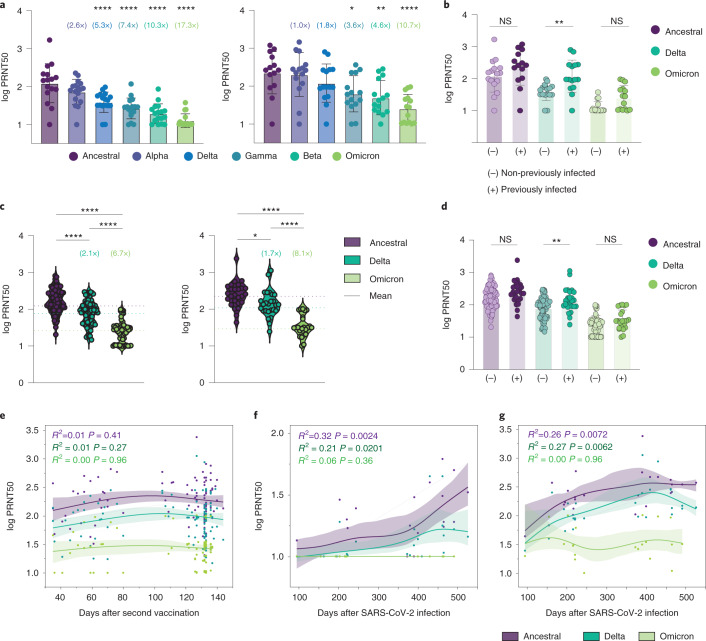
Comparison of neutralizing activity in CoronaVac–BNT162b2-vaccinated participants by SARS-CoV-2 infection status. **a**,**b**, Plasma neutralization titers against ancestral lineage A virus and variants of concern in HCW participants from Yale-New Haven Hospital who received two doses of the mRNA vaccine. **a**, Plasma neutralization titers measured at 28 days after the second dose in non-previously infected (left) and previously infected participants (right). Significance was measured using one-way ANOVA corrected using Dunnett’s test. Boxes represent the mean ± s.d. The numbers in parentheses indicate the median fold change in neutralization resistance for the indicated variants compared with the ancestral strain. **b**, Neutralization titers from the participants in **a** by SARS-CoV-2 infection status. Significance was measured using one-way ANOVA corrected using Tukey’s test. (–) Vaccinated–uninfected, *n* = 16; (+) vaccinated–previously infected, *n* = 14. **c**–**g**, Plasma neutralization titers from Dominican Republic participants who received two doses of CoronaVac followed by BNT162b2 booster. **c**, Neutralization titers against the ancestral virus and the Delta and Omicron variants at 28 days after the booster in non-previously infected (left; ancestral, *n* = 75; Delta, *n* = 75; Omicron, *n* = 75) and SARS-CoV-2-previously infected participants (right; ancestral, *n* = 26; Delta, *n* = 26; Omicron, *n* = 26). Significance was measured using one-way ANOVA corrected using Tukey’s test. Violin plots represent the mean ± s.d. Dotted lines indicate the mean and are colored accordingly. **d**, Neutralization titers from the participants in **c** by SARS-CoV-2 infection status. Significance was measured using one-way ANOVA corrected using Tukey’s test. (–) Vaccinated–uninfected, *n* = 57; (+) vaccinated–previously infected, *n* = 24. **e**, Plasma neutralization titers at 28 days after the booster dose by time from the second CoronaVac vaccination (*n* = 101 in each group). **f**,**g**, Plasma neutralization titers measured before (**f**) and 28 days after the booster dose (**g**) by time from SARS-CoV-2 infection. Ancestral, *n* = 26; Delta, *n* = 26; Omicron, *n* = 26. The lines indicate the cross-sectional average from each group and the shading represents the 95% CI. Each dot represents a single individual. A multivariable linear regression modeling was used to access significance. *P < 0.05; **P < 0.01; ***P < 0.001; ****P < 0.0001.

Next, we categorized individuals by their SARS-CoV-2 infection status (that is, previously infected versus non-previously infected) and assessed their neutralization titers against Omicron after vaccination. In individuals who had received the two mRNA vaccine doses, the neutralizing antibody titers against variants of concern for previously infected individuals were higher than those for non-previously infected individuals, 28 days after the last shot, as previously described^8^. And neutralization titers against Omicron decreased by 17.3-fold (compared with lineage A) in non-previously infected and by 10.7-fold in previously infected individuals who had received the two mRNA vaccine doses (Fig. 2a). Additionally, in individuals who received the two mRNA doses or the heterologous CoronaVac–BNT162b2 booster regimen, the neutralizing antibody titers were elevated for the Delta variant in previously infected individuals but not for Omicron (Fig. 2b–d).

**Extended Data Fig. 2:**
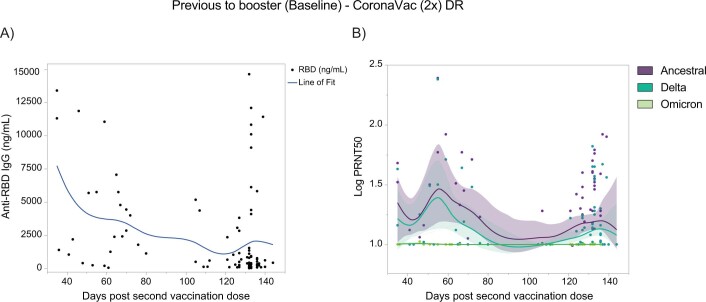
Plasma antibody kinetics post CoronaVac vaccination. **a**, Vaccinated participants plasma reactivity to RBD measured by ELISA**. b**, Plasma neutralization titers measured at baseline, previously to BNT162b2 booster shot against ancestral virus, Delta and Omicron variants. Regression lines are shown over time as days post 2 CoronaVac dose vaccination, as purple (ancestral), dark green (Delta) and light green (Omicron). Lines indicates cross-sectional averages from each group, with shading representing 95% CI and colored accordingly. Each dot represents a single individual. n = 101 (Ancestral); n = 101 (Delta); n = 80 (Omicron).

Finally, we investigated whether the timing interval between vaccination doses and SARS-CoV-2 infection correlates with vaccine-induced IgG and PRNT50 levels. As previously described^2^, anti-RBD IgG titers and neutralizing antibodies waned over time after CoronaVac vaccination (Extended Data Fig. 2). Importantly, the time interval between the second vaccination dose and the booster shot did not affect neutralization titers against ancestral and SARS-CoV-2 variants, measured 28 days after the BNT162b2 booster (Fig. 2e). However, the time interval between previous SARS-CoV-2 infection and vaccination correlated with neutralization titers against the ancestral strain and the Delta variant: the longer the time interval, the higher the neutralization responses (Fig. 2f,g). Notably, in the temporal analysis the neutralization titers against Omicron do not show such a correlation, even after the booster shot (Fig. 2g). In multivariable linear regression modeling, previous COVID-19 infection increased logPRNT50 by 0.17 (95%CI: 0.02–0.33; *P* = 0.03) for the ancestral strain, by 0.23 (95%CI: 0.08–0.39; *P* = 0.003) for the Delta variant, and by 0.15 (95%CI: −0.01 to 0.30; *P* = 0.06) for the Omicron variant after controlling for time from the second dose of the vaccine to the booster dose and for sex. It should be noted that stratification by infection status limited the sample size, which could affect the precision of our estimates for temporal analysis. Together, these data suggest that previous SARS-CoV-2 exposure improves the production of neutralizing antibodies in vaccinated individuals for the ancestral strain and the Delta variant.

We found that a regimen of heterologous two-dose CoronaVac prime vaccination followed by a single BNT162b2 booster induces elevated virus-specific antibody levels and potent neutralization activity against the ancestral and Delta SARS-CoV-2 strains, resembling the titers obtained after two doses of mRNA vaccines. After the BNT162b2 booster shot, 80% of participants developed neutralizing antibodies against Omicron and the levels were 1.4-fold higher than in participants who received two doses of mRNA vaccines. However, the neutralization titers against Omicron were significantly reduced compared with the neutralizing antibody levels against the ancestral virus and the Delta variant after the booster, suggesting a greater risk of vaccine breakthrough infections. Clinical follow-up is needed to assess the risk of serious disease in these individuals.

In agreement with previous reports, our data show that the Omicron variant escapes neutralizing antibodies elicited by two mRNA vaccine doses or two CoronaVac vaccine doses^9,12,14–16^. Notably, none of the individuals who were fully vaccinated with CoronaVac had detectable neutralizing antibodies against the SARS-CoV-2 Omicron variant. Preliminary mRNA vaccine studies suggest that booster doses can enhance neutralizing antibody response against the Omicron variant and therefore booster doses should be recommended^12,17^. However, our data suggest that the Omicron variant may be associated with lower COVID-19 vaccine effectiveness against infection, even after a single heterologous booster (CoronaVac followed by BNT162b2) and even in previously infected individuals. Our findings have immediate implications for multiple countries that previously used a two-dose regimen of CoronaVac, and are of particular importance given the increasing global need for heterologous vaccine boosters as a relevant strategy to combat the impact of emerging variants in countries where inactivated vaccines have been the dominant product used.

## Methods

### Ethics statement

This study was approved by the National Bioethics Committee of the Dominican Republic (CONABIOS). The participants received two doses of the inactivated whole-virion vaccine CoronaVac followed by a BNT162b2 booster dose at least 4 weeks after the second dose of CoronaVac. The Dominican Republic initiated the COVID-19 vaccination and booster campaigns in February and July 2021, respectively. All Dominican Republic participants provided consent to enroll in this observational study. The mRNA vaccine BNT162b2 booster was given between 30 July and 27 August 2021. HCW volunteers from the Yale-New Haven Hospital (YNHH) were enrolled and included in this study (Institutional Review Board protocol ID 2000028924, approved by the Yale Human Research Protection Program Institutional Review Board and the Yale School of Medicine). The HCW volunteers received the mRNA vaccine (100 μg mRNA-1273, Moderna or 20 μg BNT162b2, Pfizer-BioNTech) between November 2020 and January 2021. Informed consent was obtained from all enrolled. None of the participants had serious adverse effects after vaccination.

### Vaccinated volunteers

A total of 101 volunteers from the Dominican Republic and 37 HCW participants from the YNHH were followed serially after vaccination. For the Dominican Republic cohort, plasma samples were collected at baseline (before the booster, after two doses), and at 7 and 28 days after the booster (third dose). Plasma from the HCWs included in this study was collected 28 days after the second vaccination dose. Demographic information was aggregated through a systematic review and was used to construct Extended Data Tables 1 and 2. The clinical data were collected using REDCap (v5.19.15, ©2021 Vanderbilt University). Blood collection was performed and recorded by a separate team. Participants clinical information and time points of collection was not available until after the raw data had been processed and analyzed. ELISA and neutralizations were performed blinded. Prior SARS-CoV-2 infection was confirmed using ELISA and/or a polymerase chain reaction test. Documented history of vaccination and vaccination date were evidenced by the official vaccination card. The time window of previous COVID-19 infection is based on participant self-report. The time window after CoronaVac vaccination and the infection status, indicated in days post vaccination or COVID-19 exposure are available in Source Data 1.

### Plasma isolation and storage

Whole blood was collected in heparinized CPT blood vacutainers (BD Biosciences, cat. no. BDAM362780) and kept on gentle agitation until processing. All blood was processed on the day of collection in a single-step standardized method. Plasma samples were collected after centrifugation of whole blood at 600 ×*g* for 20 min at room temperature without brake. The undiluted plasma was transferred to 15 ml polypropylene conical tubes, and aliquoted and stored at −80 °C for subsequent shipping and analysis. Plasma samples were collected from Dominican Republican participants and shipped to Yale University. The plasma was aliquoted and heat-inactivated at 56 °C for 30 min to inactivate complement prior to micro-neutralization.

### SARS-CoV-2 culture

TMPRSS2-Vero E6 kidney epithelial cells were cultured in DMEM supplemented with 1% sodium pyruvate (NEAA) and 10% fetal bovine serum (FBS) at 37 °C and 5% CO_2_. The cell line had tested negative for contamination with mycoplasma. SARS-CoV-2 lineage A (USA-WA1/2020) was obtained from BEI Resources (cat. no. NR-52281). Alpha, Beta, Gamma, Delta and Omicron variants were isolated from nasopharyngeal specimens. Alpha, Beta, Gamma and Delta SARS-CoV-2 samples were sequenced as part of the Yale Genomic Surveillance Initiative’s weekly surveillance program in Connecticut, United States^17^. Omicron (lineage BA.1) was sequenced by the Connecticut State Department of Public Health (GISAID accession: EPI_ISL_7313633). The isolates were cultured and resequenced as previously described^8,18^. In brief, samples were filtered through a 45 μM filter and serially diluted from 1:50 to 1:19,531,250. The dilution was subsequently incubated with TMPRSS2-Vero E6 cells in a 96-well plate and adsorbed for 1 h at 37 °C. After adsorption, replacement medium was added, and cells were incubated at 37 °C for up to 5 days. Supernatants from cell cultures with cytopathic effect were collected, frozen, thawed and analyzed using RT–qPCR. Fresh cultures were inoculated with the lysates as described above for viral expansion. Nucleic acid was extracted using the ThermoFisher MagMAX viral/pathogen nucleic acid isolation kit and libraries were prepared using the Illumina COVIDSeq Test (RUO version). Pooled libraries were sequenced on the Illumina NovaSeq (paired-end 150) by the Yale Center for Genome Analysis. Data were analyzed and consensus genomes were generated using iVar (v1.3.1). The resequenced genomes were submitted to the National Center for Biotechnology Information (NCBI; GenBank accession numbers: ancestral lineage A, MZ468053; Alpha, MZ202178; Beta, MZ468007; Gamma, MZ202306; Delta, MZ468047; Omicron, OL965559). The pelleted virus was then resuspended in PBS and aliquoted for storage at −80 °C. Viral titers were measured by standard plaque assay using TMPRSS2-Vero E6 cells. In brief, 300 µl serial fold virus dilutions were used to infect Vero E6 cells in MEM supplemented with NaHCO_3_, 4% FBS and 0.6% Avicel RC-581. Plaques were resolved at 48 h after infection by fixing in 10% formaldehyde for 1 h followed by staining with 0.5% crystal violet in 20% ethanol. Plates were rinsed in water to plaques quantification. All experiments were performed in a biosafety level 3 laboratory with approval from the Yale Environmental Health and Safety office.

### SARS-CoV-2-specific antibody measurements

ELISAs were performed as previously described^8^. In brief, Triton X-100 and RNase A were added to serum samples at final concentrations of 0.5% and 0.5 mg ml^−1^, respectively, and incubated at room temperature for 30 min before use, to reduce the risk from any potential virus in serum. The 96-well MaxiSorp plates (Thermo Scientific, cat. no. 442404) were coated with 50 μl per well recombinant SARS CoV-2 total spike protein (ACROBiosystems, cat. no. SPN-C52H9-100 μg) or RBD (ACROBiosystems, cat. no. SPD-C52H3-100 μg) at a concentration of 2 μg ml^−1^ in PBS and incubated overnight at 4 °C. The coating buffer was removed, and plates were incubated for 1 h at room temperature with 200 μl blocking solution (PBS with 0.1% Tween-20, 3% milk powder). Plasma was diluted serially 1:100, 1:200, 1:400 and 1:800 in dilution solution (PBS with 0.1% Tween-20, 1% milk powder), and 100 μl diluted serum was added for 2 h at room temperature. Human Anti-Spike (SARS-CoV-2 Human Anti-Spike, AM006415) (Active Motif no. 91351) was serially diluted to generate a standard curve, starting at a concentration of 1,000 ng ml^−1^. Plates were washed three times with PBS-T (PBS with 0.1% Tween-20) and then 50 μl HRP anti-Human IgG Antibody (GenScript A00166, 1:5,000) diluted in dilution solution was added to each well. After 1 h of incubation at room temperature, plates were washed six times with PBS-T. Plates were developed with 100 μl TMB Substrate Reagent Set (BD Biosciences, cat. no. 555214) and the reaction was stopped after 5 min by the addition of 2 N sulfuric acid. Plates were then read at a wavelength of 450 nm and 570 nm.

### Neutralization assay

Sera from vaccinated individuals were heat treated for 30 min at 56 °C. Sixfold serially diluted plasma, from 1:10 to 1:2,430, was incubated with SARS-CoV-2 variants for 1 h at 37 °C. The mixture was subsequently incubated with TMPRSS2-Vero E6 cells in a 12-well plate for 1 h for adsorption. The cells were then overlayed with MEM supplemented with NaHCO_3_, 4% FBS and 0.6% Avicel. Plaques were resolved at 40 h after infection by fixing in 10% formaldehyde for 1 h followed by staining in 0.5% crystal violet. All experiments were performed in parallel with baseline control sera, in an established viral concentration to generate 60–120 plaques per well.

### Statistical analysis

All analyses of patient samples were conducted using GraphPad Prism 8.4.3 and JMP 15. Multiple group comparisons were analyzed by running parametric (ANOVA) statistical tests. Multiple comparisons were corrected using Tukey’s and Dunnett’s tests as indicated in the figure legends. We used a multivariable linear regression model with logPRNT50 as the dependent variable, and previous COVID-19 infection and time from the second dose of the vaccine to the booster dose (in days) as the explanatory variables to determine the effect of previous COVID-19 infection.

### Reporting Summary

Further information on research design is available in the Nature Research Reporting Summary linked to this article.

## Online content

Any methods, additional references, Nature Research reporting summaries, source data, extended data, supplementary information, acknowledgements, peer review information; details of author contributions and competing interests; and statements of data and code availability are available at 10.1038/s41591-022-01705-6.

### Supplementary information

Reporting Summary

### Source data

Source Data 1 Detailed clinical and immunological data for each patient.

## Figures and Tables

**Extended Data Table 1:**
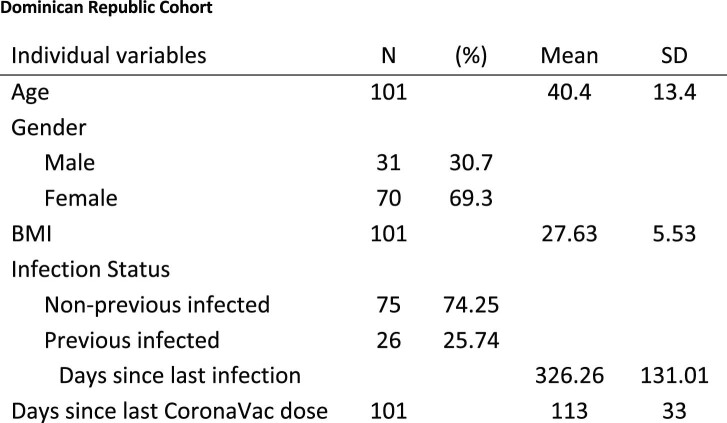
Patient data: Dominican Republic SARS-CoV-2-vaccinated cohort

Exact counts for each demographic category are displayed in N cell. Percentages of total, where applicable, are displayed in (%) cell. The mean with accompanying standard deviations for each measurement are displayed in Mean and SD cells respectively.

**Extended Data Table 2:**
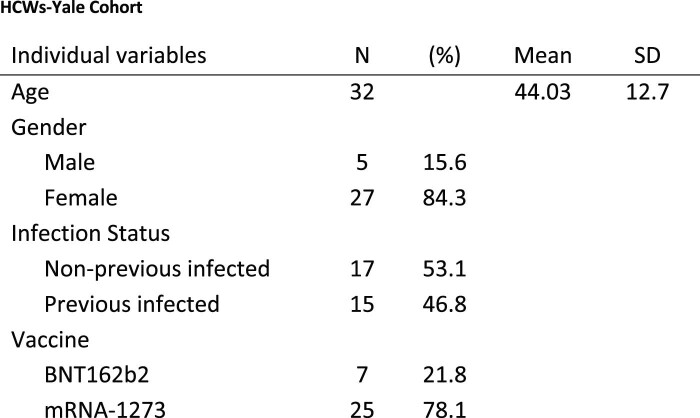
Patient data: Yale healthcare worker SARS-CoV-2-vaccinated cohort

Exact counts for each demographic category are displayed in N cell. Percentages of total, where applicable, are displayed in (%) cell. The mean with accompanying standard deviations for each measurement are displayed in Mean and SD cells respectively.

## Data Availability

All of the background participant information and data generated in this study are included in Source Data 1. The genome information and aligned consensus genomes for SARS-CoV-2 variants used in this study are available at NCBI (GenBank accession numbers: ancestral lineage A, MZ468053; Alpha, MZ202178; Beta, MZ468007; Gamma, MZ202306; Delta, MZ468047; Omicron, OL965559). Additional correspondence and requests for material should be addressed to the corresponding authors. Source data are provided with this paper.

## Extended data

are available for this paper at 10.1038/s41591-022-01705-6.
