# Supplementary material for: Neutralizing antibodies against the SARS-CoV-2 Delta and Omicron variants following heterologous CoronaVac plus BNT162b2 booster vaccination
Source: Nat Med. 2022 Jan 20;28(3):481–5. doi: 10.1038/s41591-022-01705-6 (PMC8938264; doi:10.1038/s41591-022-01705-6)
Supplement: Supplementary file 1 — Reporting Summary [file 41591_2022_1705_MOESM1_ESM.pdf]

Corresponding author(s): Akiko Iwasaki

Last updated by author(s): Jan 13, 2022

Reporting Summary

Nature Portfolio wishes to improve the reproducibility of the work that we publish. This form provides structure for consistency and transparency in reporting. For further information on Nature Portfolio policies, see our Editorial Policies and the Editorial Policy Checklist.

Statistics

For all statistical analyses, confirm that the following items are present in the figure legend, table legend, main text, or Methods section.

n/a	Confirmed	
☐	☒	The exact sample size (*n*) for each experimental group/condition, given as a discrete number and unit of measurement
☒	☐	A statement on whether measurements were taken from distinct samples or whether the same sample was measured repeatedly
☐	☒	The statistical test(s) used AND whether they are one- or two-sided<br>*Only common tests should be described solely by name; describe more complex techniques in the Methods section.*
☐	☒	A description of all covariates tested
☐	☒	A description of any assumptions or corrections, such as tests of normality and adjustment for multiple comparisons
☐	☒	A full description of the statistical parameters including central tendency (e.g. means) or other basic estimates (e.g. regression coefficient) AND variation (e.g. standard deviation) or associated estimates of uncertainty (e.g. confidence intervals)
☐	☒	For null hypothesis testing, the test statistic (e.g. *F*, *t*, *r*) with confidence intervals, effect sizes, degrees of freedom and *P* value noted<br>*Give P values as exact values whenever suitable.*
☒	☐	For Bayesian analysis, information on the choice of priors and Markov chain Monte Carlo settings
☒	☐	For hierarchical and complex designs, identification of the appropriate level for tests and full reporting of outcomes
☒	☐	Estimates of effect sizes (e.g. Cohen's *d*, Pearson's *r*), indicating how they were calculated

Our web collection on statistics for biologists contains articles on many of the points above.

Software and code

Policy information about availability of computer code

Data collection	REDCap (v5.19.15 @2021 Vanderbilt University) (clinical data aggregation).
Data analysis	Jmp Pro 15.0.0 (SAS Institute) (graphs/statistics), and GraphPad Prism 8.4.3(graphs/statistics).

For manuscripts utilizing custom algorithms or software that are central to the research but not yet described in published literature, software must be made available to editors and reviewers. We strongly encourage code deposition in a community repository (e.g. GitHub). See the Nature Portfolio guidelines for submitting code & software for further information.

Data

Policy information about availability of data

All manuscripts must include a data availability statement. This statement should provide the following information, where applicable:

- Accession codes, unique identifiers, or web links for publicly available datasets
- A description of any restrictions on data availability
- For clinical datasets or third party data, please ensure that the statement adheres to our policy

All the background information of participants and data generated in this study are included in Source Data1. The genome information and aligned consensus genomes for SARS-CoV-2 variants used in this study are available on NCBI (GenBank Accession numbers: ancestral lineage A = MZ468053, Alpha = MZ202178, Beta = MZ468007, Gamma = MZ202306, Delta = MZ468047, Omicron = OL965559). Additional correspondence and requests for materials should be addressed to the corresponding authors.

Field-specific reporting

Please select the one below that is the best fit for your research. If you are not sure, read the appropriate sections before making your selection.

☒ Life sciences ☐ Behavioural & social sciences ☐ Ecological, evolutionary & environmental sciences

For a reference copy of the document with all sections, see nature.com/documents/nr-reporting-summary-flat.pdf

Life sciences study design

All studies must disclose on these points even when the disclosure is negative.

Sample size	No statistical methods were used to calculate the sample size. Sample size was determined based on the number of adults (≥ 18 years old) from the Dominican Republic that received the mRNA vaccine, Pfizer as a booster and consent in participating in the current study. Republic initiated the COVID-19 vaccination and booster campaigns in February and July 2021, respectively. mRNA vaccine BNT162b2 booster were administrated between July 30 and August 27, 2021. This study enrolled 103 volunteers and was approved by the National Bioethics Committee of the Dominican Republic (CONABIOS). Informed consent was obtained by trained staff and sample collection commenced immediately upon study enrollment. Participants were followed serially post-vaccination. Clinical specimens were collected at baseline (previous to booster), 7- and 28- post third vaccination dose. Alpha, Beta, Gamma, Delta and Omicron variants were isolated from nasopharyngeal specimens from the Yale SARS-CoV-2 Genomic Surveillance Initiative's weekly surveillance program in Connecticut, US.
Data exclusions	Twp participant that received an adenovirus- based vaccine were excluded from this study. For the current study we had only included participants that received CoronaVac and Pfizer vaccines as booster shot.
Replication	ELISAs were done in duplicate for each sample. Neutralization assays were done with 6 fold dilution for each sample to generate a curve and access the IC50 values. For all assays, longitudinal analyses were performed from human individuals samples. All replications were successful.
Randomization	Vaccinated donors were stratified in two major groups, previously infected with SARS-CoV2 (recovered) on uninfected (naive), confirmed by serology.
Blinding	The clinical data were collected using REDCap software. Blood acquisition was performed and recorded by a separate team. Clinical information and time points of collection information was not available until after processing and analyzing raw data. ELISA and neutralizations were blinded.

Reporting for specific materials, systems and methods

We require information from authors about some types of materials, experimental systems and methods used in many studies. Here, indicate whether each material, system or method listed is relevant to your study. If you are not sure if a list item applies to your research, read the appropriate section before selecting a response.

Materials & experimental systems

n/a	Involved in the study	
☐	☒	Antibodies
☐	☒	Eukaryotic cell lines
☒	☐	Palaeontology and archaeology
☒	☐	Animals and other organisms
☐	☒	Human research participants
☒	☐	Clinical data
☒	☐	Dual use research of concern

Methods

n/a	Involved in the study	
☒	☐	ChIP-seq
☒	☐	Flow cytometry
☒	☐	MRI-based neuroimaging

Antibodies

Antibodies used	All antibodies used in this study are against human proteins:<br>Human Anti-Spike (SARS-CoV-2 Human Anti-Spike (AM006415) (Active Motif #91351) was serially diluted to generate a standard curve. HRP anti-Human IgG Antibody (GenScript #A00166, 1:5,000.
Validation	The antibodies used in this study are commercially available, and all have been validated by the manufacturers and used by other publications. Likewise, we titrated these antibodies according to our own our staining conditions.<br>Human Anti-Spike (SARS-CoV-2 Human Anti-Spike (Clone: AM006415, 415-6)) (Active Motif #91351) : this antibody has been tested by ELISA and is specific for SARS-CoV-2 Spike protein S1 Subunit of Receptor Binding Domain (RBD). Details on the development and characterization of this clone are available here: Wan J., et. al. Human IgG neutralizing monoclonal antibodies block SARS-CoV-2 infection. Cell Reports, 32: 107918, July 21, 2020.<br>HRP anti-Human IgG Antibody: this antibody is purified by immunoaffinity chromatography and then conjugated to horseradish peroxidase. Species Reactivity: human

Eukaryotic cell lines

Policy information about cell lines

Cell line source(s)	TMPRSS2-VeroE6 kidney epithelial cell line was obtained from the ATCC
Authentication	TMPRSS2-VeroE6 was obtained from ATCC, tested and authenticated by morphology, karyotyping, and PCR based approaches.
Mycoplasma contamination	The cell line tested negative for contamination with mycoplasma.
Commonly misidentified lines (See ICLAC register)	No commonly misidentified cell lines were used in the study

Human research participants

Policy information about studies involving human research participants

Population characteristics	Cohort characteristics are available in Table 1.
Recruitment	Participants were recruited between February and July 2021. mRNA vaccine BNT162b2 booster were administrated between July 30 and August 27, 2021 as part of the Republic Dominican COVID-19 vaccination booster campaigns Participants were enrollment with no self selection. Informed consent was obtained by trained staff and sample collection commenced immediately upon study enrollment. Plasma samples were collected at baseline (prior to booster, after two doses), 7 and 28 days after the booster (third dose) administration.<br>Health care worker (HCW) volunteers from the Yale New Haven Hospital (YNHH) were enrolled and included in this study. The HCWs volunteers received the mRNA vaccine (100 micrograms mRNA-1273, Moderna or 20 micrograms, BNT162b2, Pfizer-BioNTech) between November 2020 and January 2021. Plasma samples were collected at baseline (prior to vaccination, 7 and 28 days after the first and second vaccination dose. Participants were enrollment with no self selection. Informed consent was obtained by trained staff .
Ethics oversight	Yale Human Research Protection Program Institutional Review Boards. Informed consents were obtained from all enrolled healthcare workers. • Our research protocol was reviewed and approved by the Yale School of Medicine IRB Protocol ID 2000028924. This study was approved by the National Bioethics Committee of the Dominican Republic (CONABIOS). Informed consent was obtained by trained staff and records maintained in our research database for the duration of our study. There were no minors included on this study.

Note that full information on the approval of the study protocol must also be provided in the manuscript.
